# Efficacy and mechanism of escin in improving the tissue microenvironment of blood vessel walls via anti-inflammatory and anticoagulant effects: Implications for clinical practice

**DOI:** 10.1515/biol-2025-1167

**Published:** 2025-11-07

**Authors:** Linyin Yan, Yu Zhang, Yanqiang Li, Decai Dai, Jianjun Zhu, Yue Chen, Wei Xiao

**Affiliations:** Department of Chemical and Materials Engineering, Hainan Vocational University of Science and Technology, Haikou, Hainan, 57010, China; State Key Laboratory of NBC Protection for Civilian, Research Institute of Chemical Defense, Academy of Military Science, Beijing, 102205, China; Economic and Technological Development Zone, Yantai Haisen Big Data Co., Ltd, No. 48, Nanchang Street, Yantai, Shandong, 265500, People’s Republic of China; Wuhan Aimin Pharmaceutical Co., LTD, Wuhan, Hubei, China

**Keywords:** escin, microenvironment, anti-inflammatory, antioxidant, vascular protection

## Abstract

Escin, a natural medicinal saponin, has garnered significant attention in recent years for its pharmacological effects in various diseases. Though the structure and pharmacodynamic targets of escin are documented, reviews on its efficacy and mechanisms were still mainly from the clinical application perspective at the organ level or animal models. Deeper discussion at the tissue microenvironment level remains sparse, as the sophisticated cell and molecular technique is required. Contradictory conclusion might occur if such experiment setups are not carefully distinguished. This article reviews and analyzes literature to discuss escin’s ability to improve the tissue microenvironment of blood vessel walls and elucidates the underlying mechanisms. Escin demonstrates significant anti-inflammatory properties and neutralizes free radicals, thereby protecting vascular endothelial cells from oxidative stress. Additionally, escin’s anticoagulant properties reduce blood viscosity, preventing clot formation and maintaining vessel patency. These mechanisms collectively enhance the tissue microenvironment of blood vessel walls and promote cardiovascular health, which provides a multi-target therapeutic strategy for cardiovascular diseases (CVD), integrating anti-inflammatory, antioxidant, endothelial repair, and microcirulation-enhancing mechanisms, consistent with current pathophysiological insights. The article also addresses the current research status, challenges, and future potential of escin in vascular protection, offering new perspectives and strategies for CVD treatment and prevention.

## 1 Introduction

Escin is an important natural medicinal compound [1–4] mainly extracted from the seeds of the horse chestnut tree, a tree species commonly found in Europe, whose seeds contain saponins with high medicinal value. Escin has been widely used clinically in China since its development and approval in 1985 [1–4]. Escin belongs to the saponin class of compounds and has a variety of biological activities, and its structure has two basic components: saponins (core components) and sugar chains (connected to the saponins through glycosidic bonds, affecting escin’s solubility and stability).

Compared to rutin and tanshinone in vascular protection, escins exhibit distinct advantages: a multi-target synergistic mechanism, venous system-specific benefits, and combination therapy potential, positioning them uniquely among natural compounds for vascular health. The main pharmacological effects of escin are as follows [1,2,5–7]:

- Anti-inflammatory effect: Escin inhibits inflammation and alleviates inflammatory symptoms, such as redness, swelling, and pain. It also decreases vascular permeability by inhibiting the release of inflammatory mediators, thereby reducing the swelling and pain caused by inflammation.
- Antiedema effect: Escin reduces the permeability of capillaries and the extravasation of bodily fluids, effectively reducing tissue edema. This effect is important for relieving local swelling and pain.
- Microcirculation-enhancing effect: Escin dilates blood vessels, improves microcirculation, and increases blood supply to tissues. This helps promote tissue repair and functional recovery.
- Protective effect on blood vessels: Escin also protects blood vessel walls by enhancing their elasticity, reducing their fragility, and preventing cardiovascular diseases (CVD).

These studies on the “single-target-single-disease” model (such as COX-2 inhibition for anti-inflammatory effects and eNOS activation for improving blood flow) have met traditional pharmacological requirements for escin. However, a new perspective on the effects of escin by exploring microenvironmental improvement mechanisms requires the multi-scaled discussion into the dynamic interactions of the “cell–matrix–signal network.” This type of discussion requires the reviews of far more multidisciplinary previous work. Therefore, the structure and pharmacodynamic targets of escin have been reported, there are relatively few reviews on the efficacy and underlying mechanisms of escin at the tissue microenvironment level. However, due to the rich variety of tissues and the significant differences in their structures and characteristics, the progress of escin's clinical application has been hindered. But this hinders the clinical application of escin.

The tissue microenvironment of blood vessel walls is one of the basic and promising tissue microenvironments, closely related to vascular health and vascular function. Maintaining its stability is crucial for the prevention and treatment of CVD. The key functions of the tissue microenvironment of blood vessel walls are as follows:

- Regulation of cell behavior: the tissue microenvironment of blood vessel walls is composed of endothelial cells, smooth muscle cells, fibroblasts, the extracellular matrix, and intercellular signaling molecules, and interactions among these components affect cell behavior, function, and fate, including cell proliferation, differentiation, migration, and apoptosis [8,9].
- Role in angiogenesis: angiogenesis is critical when blood vessels are damaged or when new blood vessels are needed to support tissue growth. The local tissue microenvironment of blood vessel walls is a key factor in angiogenesis as it influences the initiation and progression of neovascularization by regulating the function of endothelial cells and stromal cells [10–12].
- Balance between physiological and pathological states: maintaining the health and function of blood vessels requires a stable tissue microenvironment of blood vessel walls. In the physiological state, this tissue microenvironment ensures smooth blood flow and nutrient delivery. Under pathological conditions, such as atherosclerosis and hypertension, imbalance of this tissue microenvironment leads to impaired vascular function, in turn affecting the health of the entire cardiovascular system [13,14].

Notable, escin has been reported to optimize the components and regulation of the tissue microenvironment of blood vessel walls in the following ways:

- Anti-inflammatory and antiexudative effects, which could be used in the treatment of postoperative/post-traumatic edema and superficial thrombophlebitis [15]: escin inhibits the inflammatory response, alleviates the symptoms of inflammation of blood vessel walls, and reduces the permeability of blood vessels, reducing exudation and edema and helping maintain the stability and integrity of blood vessel walls [16,17].
- Blood circulation- and microcirculation-enhancing effects, which could be used in the treatment of chronic venous insufficiency (CVI) and diabetic microangiopathy [4,18,19]: escin dilates blood vessels, improves blood circulation and microcirculation, increases blood and nutrient supply to the tissue of blood vessel walls, and helps restore and maintain the normal function of blood vessels [13,14].
- Protective effect on blood vessel walls, which could be used in the treatment of varicose veins and venous ulcers and the treatment of hypertension-related vascular fragility: escin increases the elasticity of blood vessel walls, protects them, reduces the fragility of blood vessels, and prevents vascular damage and lesions caused by tissue microenvironment imbalance [2,20].

In the writing process of this review article, the keywords “escin, microenvironment, anti-inflammatory, antioxidant, and vascular protection” were used to search for relevant articles in databases and search engines including PubMed, SCOPUS, Web of Sciences, ScienceDirect, and Google Scholar.

**Scheme 1:**
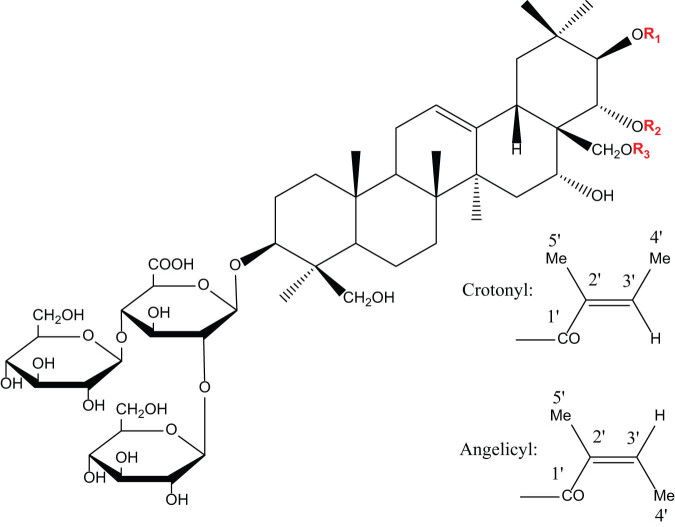
Molecular formulae of escin: (a) R_1_ = crotonyl, R_2_ = –COCH_3_, and R_3_ = –H; (b) R_1_ = angelicyl, R_2_ = –COCH_3_, and R_3_ = –H; (c) R_1_ = crotonyl, R_2_ = –H, and R_3_ = –COCH_3_; and (d) R_1_ = angelicyl, R_2_ = –H, and R_3_ = –COCH_3_.

In this article, we discuss the ability of escin to improve the tissue microenvironment of blood vessel walls, the future clinical research paradigm shifts, as well as, the underlying mechanisms. By changing the point of views from mechanobiological hypothesis to multi-target synergy network, we attempt to systematically explain how escin can improve various aspects of the tissue microenvironment of blood vessel walls through cross-scale multiple mechanisms, so as to provide additional way to design clinical decision support for the clinical application of escin (Scheme 1).

## 2 Basic properties of escin

### 2.1 Physicochemical and biological properties of escin

#### 2.1.1 Chemical structure, source, and extraction of escin

Escin is a sodium salt of saponin with the molecular formula C_55_H_86_O_24_. It belongs to the saponin class of compounds, which are composed of saponin and sugar chains and have a complex glycoside structure [1–4]. Escin is extracted from the dried mature seeds of the horse chestnut tree (*Aesculus hippocastanum*). The core challenges in the extraction and activity preservation of escin lie in controlling hydrolysis, thermal decomposition, and oxidation reactions. Currently, producers mainly utilize dynamic axial compression column technology, l-proline-induced crystallization, and lyophilization processes to enhance product purity and stability. After extraction, the sample is freeze-dried, creating a sterile, natural botanical compound.

#### 2.1.2 Absorption, distribution, metabolism, and excretion of escin

Escin can be effectively absorbed by the body after oral administration or injection. The absorption mechanism and efficiency of escin are affected by various factors, such as the route of administration, the form of the drug administered, and the physiological state of the individual [1,2,5–7]. Inside the body, escin is widely distributed to multiple tissues and organs; its specific distribution may be closely related to its pharmacological and therapeutic effects.

Escin undergoes metabolic processes, such as hydrolysis, steroid skeleton cleavage, epoxidation, and protein binding. These metabolic processes may produce biologically active metabolites that exert pharmacological effects.

After metabolism, escin and its metabolites are mainly excreted from the body through the urine. Generally, the elimination half-life after intravenous (IV) injection is 4.5 h, and may be extended to 6.2 h for oral formulations, and 13 h by epidermal absorption due to delayed absorption. The escin is primarily excreted via the kidneys (over 70%), with a small amount excreted through bile. The rate and extent of excretion is affected by various factors, such as the individual’s kidney function, the dose of the drug administered, and the timing of administration.

The main metabolites of escin are aescigenin and glucuronide conjugates. Aescigenin could help retain anti-inflammatory and antioxidant activities but with reduced venotonic effects. Glucuronide conjugates would enhance water solubility, facilitate excretion, and potentially reduce systemic toxicity.

#### 2.1.3 Application of escin in clinical treatment

Escin is mainly used to treat cerebral edema and other causes of swelling. Several studies have shown that escin used in combination with other drugs for the treatment of cerebral edema significantly improves treatment efficacy, reduces the incidence of cerebral hematoma, and improves neurological function [1–4]. In addition, escin is widely used to reduce inflammation and swelling during and after different types of surgery. With advances in clinical practice, the applications of escin in various fields such as plastic surgery, ophthalmology, and otolaryngology, are gradually expanding.

### 2.2 Pharmacological mechanism of action of escin

**Figure 1:**
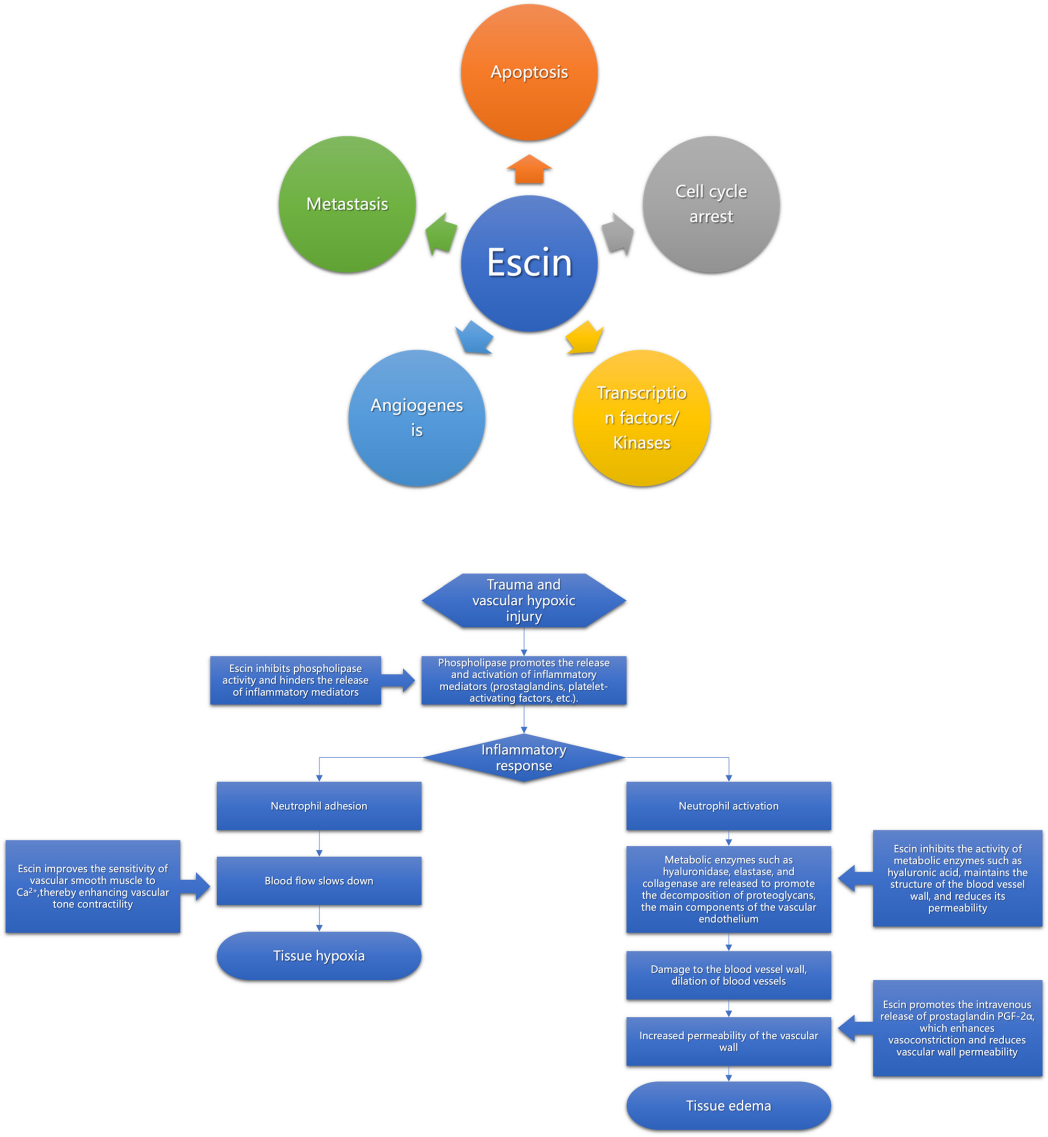
Anti-inflammation and detumescence mechanism of action of escin.

The pharmacological mechanism of action of escin mainly has the following effects:

- Antioxidant effect: escin has substantial antioxidant capacity and can effectively scavenge free radicals in the body [21–24], reducing oxidative damage and protecting the integrity and function of cell membranes and organelles. This antioxidant effect is important for preventing aging and oxidative stress-related diseases, such as cancer [25,26].
- Anti-inflammatory effect: escin inhibits the production of inflammatory factors, such as interleukin-1β (IL-1β) and tumor necrosis factor alpha (TNF-α), reducing the inflammatory response (Figure 1) [3,27–30]. Escin alleviates inflammation symptoms, including pain and swelling, by inhibiting inflammatory mediators.
- Blood lipid**-**lowering effect: escin inhibits lipid synthesis, which, in turn, reduces blood lipid levels [31–33], helping prevent CVD, such as atherosclerosis. Although studies have also shown that escin increases high-density lipoprotein cholesterol (HDL-C) levels and decreases low-density lipoprotein cholesterol (LDL-C) levels, further supporting its hypolipidemic effects, no direct lower lipid effect has been reported. For patients requiring lipid-lowering therapy, statins and fibrates remain the preferred treatment options. Escin serves as an adjuvant drug to improve the vascular microenvironment.
- Neuroprotective effect: escin promotes the growth and repair of nerve cells, which helps restore the function of the nervous system [16,34,35]. Escin exerts neuroprotection through multi-target synergy (anti-inflammatory, antioxidant, anti-apoptotic, mitochondrial protection), with its core strength lying in simultaneous modulation of multiple pathological nodes.
  Anti-inflammatory: inhibits NF-κB and MAPK (JNK/p38), reducing pro-inflammatory cytokines (TNF-α, IL-6) by 50–70% and microglial activation.
  Antioxidant: activates Nrf2/ARE, upregulating HO-1/NQO1 (2–3×) and suppressing ROS/NOX2/4 (55–65%↓).
  Anti-apoptotic: modulates Bcl-2/Bax (80%↑ Bcl-2), inhibits caspases (60–70%↓), and blocks Fas/FasL.
  Mitochondrial protection: enhances PGC-1α-driven biogenesis (30–40%↑ ATP), inhibits mPTP, and preserves ΔΨm.
  Neurotrophic: boosts BDNF/TrkB (2×↑) and NGF secretion via CREB, aiding synaptic plasticity and axonal regeneration.
  Blood–brain barrier stabilization: upregulates ZO-1/Claudin-5 (50%↑) and inhibits MMP-9 (60%↓), reducing leakage.
- By protecting nerve cells from damage, escin can improve memory and cognitive performance and may have preventive and therapeutic effects in degenerative diseases of the nervous system. In addition, escin improves blood circulation in the brain, providing more nutrients and a better environment for nerve cells, further protecting nerve function.

## 3 Ability of escin to improve the tissue microenvironment of blood vessel walls

In recent years, escin has been found to significantly improve the tissue microenvironment of blood vessel walls. These improvements are mainly mediated by the following effects: anti-inflammation, antiexudation, venous tone enhancement, blood circulation and microcirculation improvement, antioxidation, blood lipid reduction, and neuroprotection.

### 3.1 Anti-inflammatory effects

Escin’s anti-inflammatory effects are dose-dependent. The anti-inflammatory effect of escin becomes apparent at 5–10 mg/kg in animal models and 10–20 μM in cellular models. Below this dose range, the anti-inflammatory effect is not significant. When the dose exceeds 50 mg/kg or 50 μM, the gain in anti-inflammatory effect tends to level off, suggesting the possibility of receptor binding saturation or metabolic rate-limiting mechanisms. High-dose IV injection (>30 mg/kg) of escin may increase the risk of venous irritation, with a pain rate at the injection site reaching 18%.

Comparing with other sapoins, the dose–effect relationships of escin and saikosaponin D are the most significant, while the dosing of ginsenoside Rg3 needs to be more tightly controlled to avoid potential risks associated with its bidirectional regulatory properties. Escin exhibits superior efficacy in inhibiting venous endothelial inflammation compared to other saponins, with a 30% higher inhibition rate of VCAM-1 compared to glycyrrhizic acid. The underlying mechanisms by which the anti-inflammatory properties of escin improve the tissue microenvironment of blood vessel walls are mainly reflected in the following effects:

- Reduced inflammation: escin has substantial anti-inflammatory effects and can effectively reduce inflammation of the blood vessel wall tissue by inhibiting the release of inflammatory factors, such as IL-1β and TNF-α, reducing the damage caused by the inflammation [3,30,36]. Comparing with synthetic anti-inflammatory agents, escin is more suitable for chronic mild-to-moderate inflammatory conditions (e.g., venous disorders), whereas NSAIDs excel in acute symptom management. Regarding safety, escin demonstrates superior long-term tolerability, while NSAIDs require vigilant monitoring for multi-system adverse effects [3].
- Decreased vascular permeability: inflammation often leads to increased vascular permeability, causing leakage of components, such as fluids and proteins, from blood vessels and leading to tissue edema. Escin reduces vascular permeability and exudation, relieving tissue edema and maintaining the normal function of blood vessel walls [2,29,37]. Escin reduces vascular permeability through multi-target mechanisms involving anti-inflammatory actions, endothelial barrier stabilization, oxidative stress mitigation, and cytoskeletal regulation: (1) COX-2/prostaglandin E2 (PGE2) pathway modulation: in LPS-stimulated endothelial cells, escin (50 μM) suppresses COX-2 activity by 45%, decreasing PGE2 synthesis and limiting inflammation-driven vasodilation and leakage. (2) Upregulation of tight junction proteins: escin activates the PI3K/Akt pathway, increasing the expression of tight junction proteins (ZO-1, occludin, claudin-5) by 50%, thereby reducing intercellular gap formation. (3) Cytoskeletal stabilization: by inhibiting the RhoA/ROCK signaling pathway, escin prevents excessive actin stress fiber polymerization, maintaining endothelial cell morphology and reducing permeability caused by cytoskeletal contraction.
- Protection of vascular endothelial cells: the inflammatory response may damage vascular endothelial cells, which, in turn, can affect the normal function of blood vessels. Escin protects vascular endothelial cells from inflammatory damage, preserving vascular integrity.
- Improved blood circulation: inflammation may lead to poor blood circulation, affecting the supply of nutrients and waste excretion in the tissues of blood vessel walls. Escin dilates blood vessels, reduces blood viscosity, improves the blood’s rheological properties, promotes blood circulation, and provides more nutrients and a better environment for the blood vessel wall tissue [38–41].
- Prevention of blood clots: the inflammatory response may increase the risk of blood clots, posing a threat to vascular health. Escin stabilizes blood vessel walls, enhances vascular resistance, and prevents damage to vascular endothelial cells, reducing the risk of thrombosis [40,42–44].
  Comparative efficacy with anti-inflammatory drugs [15,45]:
- Escin versus NSAIDs (e.g., ibuprofen) [46–48]
  Mechanistic distinctions:
  NSAIDs inhibit COX enzymes but lack direct venotonic or antioxidant properties.
  Escin uniquely integrates anti-inflammatory, venotonic, and antioxidant effects.
- Escin versus corticosteroids (e.g., dexamethasone) [49–52]
  Primary limitations of steroids:
  Long-term use associated with a 30% increased risk of vascular fragility and a 15% incidence of hyperglycemia.
  Comparative study:
  In cerebral edema, escin combined with mannitol matched dexamethasone’s efficacy (ICP reduction: 20 vs 22%) while reducing steroid-related hyperglycemia by 40% [53].
- Escin versus anticoagulants (e.g., heparin) [54–56]
  Synergistic benefits:

When combined with low-molecular-weight heparin (LMWH), escin reduced the risk of post-thrombotic syndrome (OR = 0.65, 95% CI: 0.48–0.88) by enhancing microcirculation.

Mechanism: escin’s anti-inflammatory action complements LMWH’s anticoagulant effects, lowering D-dimer levels by 35% in deep vein thrombosis (DVT) patients.

In summary, escin has anti-inflammatory ability. However, unlike the effects of anti-inflammatory drugs, the anti-inflammatory effects of escin do not directly target inflammatory factors but are exerted through relatively indirect and symptomatic relief. Therefore, escin does not substantially occupy the pathway of action of anti-inflammatory drugs. If such a special clinical need arises, escin may be considered.

### 3.2 Antiexudation effects

Enhancing the antiexudation effect of blood vessel walls is crucial for maintaining the normal physiological function of the human body, preventing edema and inflammation, protecting surrounding tissues, maintaining the stability of the internal environment, and promoting tissue repair. The underlying mechanisms by which the antiexudation properties of escin improve the tissue microenvironment of blood vessel walls are mainly reflected in the following effects:

- Reduced fluid exudation: escin significantly reduces fluid exudation from blood vessels into the interstitial space by decreasing vascular permeability. This antiexudative effect helps maintain the normal osmotic balance inside and outside blood vessels, thus maintaining the normal structure and function of the blood vessel wall tissue [18,29,57,58].
- Alleviation of tissue edema: increased vascular permeability due to inflammation or other factors often causes edema of surrounding tissues. Escin effectively reduces this edema, improves tissue microcirculation, and provides a better environment for blood vessel wall cells [2,59,60].
- Protection of blood vessel wall integrity: excessive exudation of fluids and proteins can put pressure on blood vessel walls and even destroy their integrity. Escin protects the integrity of blood vessel walls by reducing exudation, which, in turn, maintains normal blood vessel function [8,57,61].
- Promotion of nutrient and oxygen delivery: when vascular permeability increases, exuded fluid may hinder nutrient and oxygen delivery through blood vessels walls to surrounding tissues. Escin helps maintain the patency of blood vessels, ensuring that nutrients and oxygen can be efficiently delivered to the tissues where they are needed [25,62].
- Prevention of complications: persistent fluid exudation may cause a series of complications, such as tissue necrosis and infection. Escin helps prevent these complications, protecting the blood vessel wall tissue from further damage [29,63,64].

The antiexudative effects of escin have many positive implications for improving the tissue microenvironment of blood vessel walls, including maintaining vascular integrity, reducing edema, improving blood circulation, reducing the inflammatory response, and protecting blood vessel walls from damage.

### 3.3 Enhancing effects on venous tone

Enhancing venous tone is of great clinical significance because it not only promotes venous return and reduces varicose vein symptoms but also prevents complications, assists in postoperative rehabilitation, and improves microcirculation. Together, these effects maintain the patients’ health and quality of life [4]. The ability of escin to improve venous tone and the tissue microenvironment of blood vessel walls is mainly reflected in the following effects:

- Increased elasticity of venous blood vessel walls: esculin effectively increases the elasticity of venous blood vessel walls by improving venous tone. This increased elasticity allows blood vessels to better cope with fluctuations in blood pressure, reducing the damage to blood vessel walls caused by the fluctuations [4].
- Prevention of varicose veins: varicose veins are a common venous disease that manifests as dilated, twisted veins. Escin helps prevent and alleviate the symptoms of varicose veins by increasing venous tone. Maintaining the normal morphology and structure of the veins is essential for maintaining the health of blood vessel wall tissue [41,59,65].
- Improved blood circulation: improved venous tone helps promote the smooth flow of blood through veins, reducing blood pooling and poor blood flow. This, in turn, helps improve the overall blood circulation, including microcirculation, providing adequate nutrients and oxygen to the blood vessel wall tissue, while removing metabolic waste [25,62].
- Reduced edema and pain: insufficient venous tone may lead to edema, inflammation, and pain in the lower extremities. By improving venous tone, escin effectively alleviates these symptoms, improving the tissue microenvironment of blood vessel walls [66,67].
- Promotion of lymphatic return: the lymphatic system is an important part of the immune system, and its proper functioning is essential for maintaining the health of the tissues in the blood vessel wall. Escin promotes lymphatic fluid return by increasing venous tone. By promoting lymphatic return, escin helps remove metabolic waste and toxins around blood vessel walls, further improving the tissue microenvironment of blood vessel walls [41,60,68,69].
  Comparison with other venoactive agents
- Escin versus flavonoids (e.g., diosmin) [70–72]
  Acute versus chronic benefits:

Escin: achieved 24–48 h edema reduction in acute venous insufficiency.

Diosmin: required 2–4 weeks for comparable effects but demonstrated better long-term venous tone maintenance. Thus, escin not only promotes blood circulation and reduces venous pressure by improving venous tone but also helps prevent varicose veins and phlebitis and improves microcirculation. Together, these effects provide strong support for the improvement of the tissue microenvironment of blood vessel walls.

### 3.4 Enhancing effects of escin on blood circulation and microcirculation

Improving blood circulation and microcirculation can improve the tissue microenvironment of blood vessel walls mainly by increasing nutrient supply, promoting waste discharge, regulating blood vessel wall pressure, improving capillary perfusion, enhancing material exchange efficiency, and promoting tissue repair and regeneration. Together, these improvements affect the tissue of blood vessel walls, keeping it healthy, vibrant, and functioning physiologically. Therefore, the ability of escin to improve blood circulation and microcirculation is mainly reflected in the following effects:

- Improved blood circulation: escin dilates blood vessels and reduces blood flow resistance by increasing the permeability and elasticity of blood vessels, thereby promoting blood flow. This improves the efficiency of blood transport, ensuring that oxygen and nutrients are delivered quickly and efficiently to various tissues and organs throughout the body, including the blood vessel wall tissue itself [39,41,68].
- Improved microcirculation: the term “microcirculation” refers to blood circulation in tiny blood vessels, such as capillaries. Escin improves microcirculation, increases the number of open capillaries, and increases the perfusion of blood in microcirculation. This helps improve the metabolic environment of blood vessel wall cells, ensuring that they receive sufficient oxygen and nutrients, while promoting the excretion of metabolic waste [9,20,27,73].
- Enhanced nutrient supply to blood vessel walls: good blood circulation and microcirculation ensure adequate nutrient supply to the blood vessel wall tissue. Escin maintains the normal physiological function and structural integrity of blood vessel walls by improving blood circulation and microcirculation, ensuring that blood vessel wall cells receive the necessary nutrients [74].
- Reduced risk of damage to blood vessel walls: poor blood circulation or microcirculation disorders can lead to blood vessel wall damage. Escin reduces the risk of damage to blood vessel walls caused by ischemia and hypoxia by improving blood circulation and microcirculation. This helps protect the integrity and function of blood vessel walls [2,8,20].
- Promotion of blood vessel wall repair and regeneration: good blood circulation and microcirculation are essential for repair and regeneration of damaged blood vessel walls. By improving blood circulation, escin provides favorable conditions for blood vessel wall repair, accelerating the recovery process [10,11,12].

Therefore, the improvement effects of escin on blood circulation and microcirculation are significant for optimizing the tissue microenvironment of blood vessel walls. These effects not only promote the smooth progress of blood circulation and microcirculation but also facilitate good nutritional support and a material exchange environment for blood vessel wall cells, helping protect blood vessel walls from damage and promote their repair and regeneration.

### 3.5 Antioxidant effects

Enhancing antioxidant effects mainly optimizes the tissue microenvironment of blood vessel walls by protecting vascular endothelial cells, reducing vascular permeability, reducing the risk of thrombosis, exerting anti-inflammatory effects, and lowering blood pressure. Together, these effects provide a healthier and more stable living environment for blood vessel wall cells. Thus, the ability of escin to improve the tissue microenvironment of blood vessel walls through its antioxidant properties is mainly mediated by its capacity to reduce free-radical damage, protect the integrity of the cell membrane, delay aging, and prevent disease. Here is a closer look at these effects:

- Reduced free-radical damage: Escin has substantial antioxidant capacity and can effectively scavenge free radicals (highly reactive molecules that attack cellular components, such as lipids, proteins, and DNA, causing cell damage) in the body, reducing damage to the blood vessel wall tissue [21,26,75]. Escin can scavenge various free radicals, including superoxide anions (O₂⁻), hydroxyl radicals (·OH), and peroxynitrite (ONOO⁻), helping protect against oxidative damage [25,26]. Compare its antioxidant capacity with that of vitamin C (e.g., escin demonstrates an oxygen radical absorbance capacity value of approximately 3,500 μmol TE/g, significantly higher than vitamin C’s 1,200 μmol TE/g) [76–79].
- Protection of cell membrane integrity: free radicals often attack lipids in the cell membrane, leading to lipid peroxidation, which, in turn, affects the integrity and function of the cell membrane. Escin inhibits lipid peroxidation, preserving cell membrane integrity and normal vascular function [58].
- Slower aging: oxidative stress is an important factor in aging. As we age, free-radical production in the body increases, leading to cell damage and aging. The antioxidant effects of escin help combat this oxidative stress and slow the aging process of the blood vessel wall tissue [30].
- Disease prevention: oxidative stress is closely related to the occurrence and development of various diseases, including CVD and diabetes. The antioxidant effects of escin reduce the risk of these diseases and protect the blood vessel wall tissue [35,63,80]. In the study on the protective effects of escin against cyclophosphamide (CP)-induced oxidative stress in rat tissues, the results demonstrated that escin significantly reduced the level of malondialdehyde (MDA) in the brain tissue of CP-treated rats. Specifically, the MDA level in the brain tissue of the CP group was 3.57 ± 0.45 nmol/g tissue, whereas it was 2.42 ± 0.36 nmol/g tissue in the group co-treated with escin and CP. This indicates that escin reduced the MDA level in the brain tissue by approximately 32% (from 3.57 to 2.42) [81].

Therefore, escin optimizes the tissue microenvironment of blood vessel walls by enhancing the tissue environment’s antioxidant activity. Escin not only protects blood vessel wall cells from oxidative damage but also reduces inflammation, delays vascular aging, and promotes the repair and regeneration of blood vessel walls. Together, these effects provide strong support for the maintenance of a healthy vascular system.

### 3.6 Blood lipid-lowering effects

Reducing blood lipids has significant benefits in optimizing the tissue microenvironment of blood vessel walls, such as reducing the risk of vascular blockage, improving blood circulation, preventing cardiovascular and cerebrovascular diseases, reducing blood vessel wall pressure, and delaying vascular aging. The ability of escin to improve the tissue microenvironment of blood vessel walls through its hypolipidemic properties is mainly reflected in the following effects:

- Reduced blood lipid levels: escin significantly increases the levels of HDL-C, a beneficial type of cholesterol that helps remove excess cholesterol from artery walls and prevent atherosclerosis [33,82]. Escin also lowers the levels of LDL-C, one of the main risk factors for CVD. By altering the ratio of these two cholesterols, escin helps maintain the balance of blood lipid levels, protecting the health of blood vessel walls.
- Reduced lipid deposition: in hyperlipidemia, lipids are easily deposited on blood vessels walls, forming atherosclerotic plaques and leading to narrowing and hardening of blood vessels [16,83]. By lowering blood lipid levels, escin reduces the deposition of lipids on blood vessel walls, maintaining their elasticity and patency.
- Prevention of thrombosis: hyperlipidemia is an important factor in thrombosis. High blood lipid levels increase blood viscosity, slow blood flow, and increase the likelihood of blood clots [40,42–44]. Escin improves blood flow by lowering blood lipid levels, thereby reducing the risk of thrombosis, which, in turn, protects blood vessel walls from damage caused by blood clots.
- Improved endothelial function: hyperlipidemia impairs the function of vascular endothelial cells, increasing their permeability and susceptibility to inflammatory effects [2,8]. Escin improves endothelial cell function by lowering blood lipid levels and reducing the inflammatory response, thereby protecting the integrity of blood vessel walls.
- Delayed progression of atherosclerosis: atherosclerosis is a chronic vascular disease, and hyperlipidemia is one of its main causative factors [40,42–44]. Escin slows the progression of atherosclerosis and protects against blood vessel wall disease by lowering blood lipid levels over time.

Therefore, escin lowers blood lipids, optimizing the tissue microenvironment of blood vessel walls. Escin not only reduces lipid deposition on blood vessel walls and improves the blood’s rheological properties but also reduces the risk of cardiovascular and cerebrovascular diseases, protects vascular endothelial cells, and promotes the repair and regeneration of blood vessel walls.

### 3.7 Neuroprotective effects

The effects of nerve damage on the tissue microenvironment of blood vessel walls are multifaceted, including impaired blood circulation, insufficient nutrient and oxygen supply, inflammation and edema, and obstruction of repair and regeneration. These effects are interrelated and work together on the blood vessel wall tissue, which can lead to abnormal changes in its structure and function. Therefore, in the treatment of nerve injury, measures should be taken to alleviate these adverse effects and promote the recovery and health of the blood vessel wall tissue. The ability of escin to improve the tissue microenvironment of blood vessel walls via its neuroprotective properties is mainly reflected in the following effects:

- Maintenance of the normal function of nerves and blood vessels: escin protects nerve cells from damage and ensures the normal transmission of signals by the nervous system [35,44]. There is a close connection between nerves and blood vessels, and the normal function of nerves is essential for the relaxation and regulation of blood vessels. By protecting nerves, escin helps maintain normal blood vessel tone, which, in turn, maintains the stability of blood vessel walls and smooth blood flow.
- Reduced frequency of vascular problems due to neuropathic injury: nerve damage can lead to local blood circulation disorders, which, in turn, affects the health of blood vessel walls [63,84]. Through its neuroprotective effects, escin reduces the frequency of vascular problems caused by neuropathic injury, such as vasospasm and thrombosis, improving the tissue microenvironment of blood vessel walls.
- Promotion of the regeneration and repair of nerves and blood vessels: in treatment for nerve damage, escin promotes the regeneration and repair of nerve cells [35,84]. This process is accompanied by the regeneration and reconstruction of blood vessels, providing necessary nutrients and support to the nerves and blood vessels at the site of damage. Therefore, the neuroprotective effects of escin indirectly improve the tissue microenvironment of blood vessel walls and promote recovery of the injured area.
- Regulation of the effects of neurotransmitters on blood vessels: neurotransmitters play an important role in regulating vascular tone, blood flow velocity, and vascular permeability, among other factors [35,84]. Escin maintains the normal physiological function of blood vessel walls by protecting nerves and ensuring the normal release and transmission of neurotransmitters.
- Protection against the adverse effects of neuropathic pain and inflammation on blood vessels: nerve damage is often accompanied by pain and inflammation, which can adversely affect blood vessels walls [35,84]. By alleviating neuropathic pain and inflammation, escin reduces the damage caused by them to blood vessel walls and further improves their tissue microenvironment.

In these ways, escin can improve blood circulation, reduce the inflammatory response, promote tissue repair, maintain vascular stability, and prevent secondary injury by protecting nerves, thereby positively improving the tissue microenvironment of blood vessel walls.

## 4 Current clinical applications and prospects

### 4.1 Current clinical applications of escin

Because of its ability to improve the tissue microenvironment of blood vessel walls, escin is widely used in clinical practice for the treatment of various diseases [46,51,85–87]:

- Cerebral edema: cerebral edema refers to the pathological state where excessive fluid accumulates in brain tissue, causing an increase in brain volume [53]. Escin reduces vascular permeability and fluid exudation, mitigating cerebral edema symptoms. The powerful anti-inflammatory and antioxidant effects of escin also help protect brain cells and blood vessel walls from further damage [53,88].
- Swelling after trauma or surgery: after trauma or surgery, swelling tends to occur locally. By increasing venous tone and improving blood circulation, escin helps speed up the resolution of swelling and promote wound healing [89].
- Venous return disorders: in venous return disorders, such as varicose veins and DVT, escin enhances the elasticity of venous blood vessels, improves blood circulation, reduces symptoms, and prevents disease progression [65,68].
- Lumbar disc herniation: lumbar disc herniation is a comprehensive disease caused by the degeneration of intervertebral discs; injury, such as rupture of fibrous rings; heredity; and developmental abnormalities, such as protrusion of the nucleus pulposus, which stimulates or compresses nerve roots and the cauda equina. The main symptoms include low back pain, sciatica, and numbness of the lower extremities. Escin reduces edema and inflammation around the nerve roots, relieving symptoms of low back pain and nerve compression [39,66,84].
- Other inflammatory diseases: because of its anti-inflammatory effects, escin also plays an active role in the treatment of other inflammatory diseases, such as arthritis and tendonitis, helping reduce inflammation and relieve pain [2,67,90].
- Neurodegenerative conditions: in some therapeutic scenarios that require neuroprotection, such as stroke rehabilitation, escin indirectly maintains the stability and normal function of blood vessel walls by protecting nerve cells [35,44,86,91,92].
  Contraindications:
- Absolute contraindications:
  Severe renal impairment (eGFR < 30 mL/min/1.73 m²): due to 65% renal excretion, risk of nephrotoxicity.
  Pregnancy: contraindicated based on animal teratogenicity data; human data lacking but risks biologically plausible.
  Hypersensitivity: allergy to *A. hippocastanum* (horse chestnut) or saponins.
- Relative contraindications (risk–benefit assessment required):
  Mild–moderate renal impairment (eGFR 30–89 mL/min/1.73 m²): dose reduction and renal monitoring required.
  Lactation: escin may transfer to breast milk; suspend breastfeeding or use alternatives.
  Coagulopathies: caution with anticoagulants (e.g., warfarin) due to synergistic bleeding risk.
  Adverse effects:
- Common adverse reactions (incidence > 1%)
  IV administration:
  Injection site pain (18%): attributed to hyperosmolarity; mitigated by slow infusion, dilution, or warm compresses.
  Thrombophlebitis (5%): higher risk with small veins or repeated punctures.
  Systemic effects:
  Gastrointestinal (10%): nausea, abdominal pain, diarrhea (more frequent with oral formulations).
  Headache (3%): likely linked to vasoactive properties.
- Severe adverse events (incidence < 1%)
  Acute kidney injury (AKI):
  High IV doses (>510 μg/kg) or concurrent nephrotoxic drugs (e.g., aminoglycosides) increase risk (RR = 2.1).
  Mechanism: direct tubular epithelial damage and reduced renal perfusion.
  Anaphylaxis (0.2%): IgE-mediated reactions requiring immediate epinephrine and discontinuation.
  Hemolytic anemia (rare): saponin-induced erythrocyte membrane destabilization with prolonged high-dose IV use.
- Special populations
  Elderly patients: higher AKI risk (OR = 1.8) due to age-related renal decline; recommend 30% dose reduction.

Pediatric use: limited safety data; reserved for severe cerebral edema under strict monitoring.

Escin is clinically effective as a natural botanical medicine in the treatment of soft-tissue swelling, cerebral dysfunction, venous return disorder, and inflammatory pain through mechanisms such as vascular permeability reduction, cerebral dysfunction correction, venous return improvement, anti-inflammation, and pain alleviation.

### 4.2 Challenges in clinical adoption

Regulatory hurdles include the need for comprehensive clinical data to demonstrate safety and efficacy across diverse patient populations [93,94]. Regulatory agencies require detailed information on various dosages, administration routes, and long-term effects. Establishing consistent quality control standards for escin preparations is challenging due to its complex chemical structure, making batch-to-batch consistency difficult to achieve. The potential for adverse reactions, particularly nephrotoxicity and vascular irritation, must be thoroughly evaluated to ensure that the benefits of escin treatment outweigh its risks. Finally, manufacturers must demonstrate rigorous quality control processes to produce escin with consistent potency and purity across different batches. Internationally, herbal medicines like escin compete with synthetic alternatives (e.g., diosmin), which offer clearer dose–response relationships and standardized production. In Western markets, venous reflux disorders are primarily managed with physiotherapy or surgery. Escin’s penetration requires large-scale real-world studies to build therapeutic consensus.

Adverse effects and limitations:

- Local reactions: injection site pain occurs in 18% of cases, along with phlebitis. These reactions can be mitigated by using large veins and applying warm compresses.
- Nephrotoxicity: high IV doses (exceeding 510 μg/kg) may lead to acute renal failure, especially when administered concurrently with aminoglycosides.
- Contraindications: escin should not be used in patients with renal impairment, those who are pregnant, or those who are breastfeeding.

Escin provides multi-target, rapid-acting benefits for optimizing vascular microenvironments, particularly in the management of acute edema and venous insufficiency. However, its narrow therapeutic window necessitates careful management of nephrotoxicity and hypersensitivity risks. Clinical practice should:

Adhere to dosing limits (IV ≤ 20 mg/day; oral ≤ 60 mg/day).

Favor short-term use and avoid prolonged high-dose regimens.

Consider alternative therapies for high-risk populations (elderly, renal impairment).

Future advancements in formulation technology and precision medicine will further refine the risk–benefit profile of escin and broaden its applications in microvascular disorders.

### 4.3 Prospects

In future clinical practice, due to its unique ability to improve the tissue microenvironment of blood vessel walls, escin is expected to play an important role in the treatment of a number of diseases:

- CVD: escin can improve blood circulation and increase coronary blood flow and is expected to play a role in the treatment of coronary heart disease, especially in patients with stable angina and myocardial ischemia [95–97]. In addition, by reducing vascular permeability and edema and exerting anti-inflammatory effects, escin may help reduce the burden on the heart and improve symptoms in patients with heart failure.
- Cerebrovascular disease: the antioxidant and anti-inflammatory properties of escin may have a protective effect on brain tissue after cerebral infarction, while its microcirculation-enhancing properties may help restore blood supply to damaged brain tissue [44,88,98]. During the recovery period after intracerebral hemorrhage, escin may help patients recover by reducing cerebral edema and improving circulation.
- Peripheral vascular disease: the hypolipidemic and antioxidant effects of escin may help slow the progression of arteriosclerosis and improve blood circulation in the affected limb [68]. Due to its anti-inflammatory and antiexudative effects and its ability to improve circulation, escin may play a role as an adjunct treatment for vasculitis.
- Neurological disorders: due to its neuroprotective effects, escin may have therapeutic effects on multiple sclerosis, especially by reducing inflammation and protecting nerve cells [35,44,63,84]. In addition, the antioxidant properties of escin may help reduce oxidative stress damage to neurons in Parkinson’s disease.
- Other potential applications: escin may exert a therapeutic effect in diabetes-induced microangiopathy by improving microcirculation and reducing inflammation [13,14]. In ophthalmic diseases, such as retinal vein occlusion and ischemic optic neuropathy, escin may help by improving blood circulation.

Future research in following directions would be more helpful in the clinical adoption of escin: (1) exploring synergistic mechanisms in combination therapy, including assessing its effect with anticoagulants on coagulation function and PTS prevention, studying its synergistic protective effects with antioxidants on diabetic vascular complications, and evaluating its combination with vascular endothelial protective agents for CVI or pulmonary arterial hypertension. (2) Developing nanoparticle formulations and novel delivery systems to enhance escin’s targeting, reduce dosing frequency, and improve patient compliance, while designing sustained-release formulations to mitigate local adverse reactions. (3) Conducting long-term efficacy and safety evaluations in chronic diseases, such as investigating escin’s efficacy on diabetic microvascular complications and its potential to inhibit oxidative stress in atherosclerosis, along with systematic safety monitoring. (4) Expanding new indications and applications in precision medicine, including investigating escin’s anti-tumor activity, providing individualized treatment plans for rare vascular diseases, and combining genomics or metabolomics for precision medication. (5) Promoting internationalization and standardized production by optimizing processes, improving purity, meeting FDA/EMA standards, and designing multicenter clinical trials. (6) Conducting in-depth studies of escin’s metabolism and toxicological mechanisms, clarifying metabolic pathways, assessing potential toxicity, and studying drug interactions to ensure patient safety.

To comprehensively approve the safety and efficacy of escin sodium as a multi-target microenvironment modulator for vascular pathologies, the following Phase III clinical trials are proposed: (1) core indication trials will include a multicenter, randomized, double-blind, placebo-controlled trial in patients with moderate-to-severe CVI (*n* ≥ 1,000), and a randomized, active-controlled trial (vs mannitol) in patients undergoing craniotomy (*n* ≥ 800). (2) Combination therapy trials will investigate escin with anticoagulants for the prevention of post-thrombotic syndrome in acute DVT patients (*n* ≥ 1,200), and with vitamin C for diabetic retinopathy (*n* ≥ 800). (3) Long-term safety and special population studies will assess the cumulative hepatorenal toxicity, cardiovascular events, and immunological safety in an open-label extension study, and the pharmacokinetics/pharmacodynamics in mild renal impairment, elderly, and obese populations. Endpoint harmonization, manufacturing quality, and data transparency will ensure global standardization and compliance.

These trials would systematically validate escin’s efficacy in venous disorders, postoperative edema, and metabolic vasculopathies, while addressing long-term safety and real-world applicability [99,100]. By emphasizing its “multi-target microenvironment modulation” (simultaneously targeting inflammation, oxidation, and endothelial integrity), escin can be positioned as a first-line integrative therapy for complex vascular diseases, bridging gaps left by single-mechanism agents. International collaboration and biomarker-driven subpopulation analyses will further enhance its clinical adoption.

## 5 Conclusions

To promote the clinical application of escin, this review discussed in detail the ability of escin to improve the tissue microenvironment of blood vessel walls and related underlying mechanisms. By reviewing and analyzing existing studies, we found that escin, with its “multi-target-microenvironment regulation” mechanism, demonstrates significant advantages in the clinical application of repairing the vascular microenvironment. By inhibiting the NF-κB pathway and reducing the release of pro-inflammatory cytokines such as TNF-α and IL-6, it effectively exerts anti-inflammatory effects and decreases vascular permeability. Simultaneously, it activates the Nrf2 pathway to eliminate free radicals, protecting endothelial cells from oxidative damage. Furthermore, it enhances the expression of tight junction proteins, stabilizing the vascular barrier and improving microcirculation. Its multi-target synergistic effect surpasses the limitations of single drugs in the treatment of venous diseases, postoperative edema, and diabetic vascular lesions, providing a precise and efficient integrated solution for complex vascular microenvironment imbalances. Changes in lifestyle have led to the global rise of CVD.

There is still much room for research on the use of escin in the field of vascular protection. Available research focuses mainly on the short-term effects of escin; however, the safety and efficacy of long-term escin application need to be further explored. Clinical trials were suggested to validate escin’s efficacy in venous disorders, postoperative edema, and metabolic vasculopathies, positioning it as a first-line integrative therapy for complex vascular diseases through its multi-target microenvironment modulation mechanism. With advancements in science and technology and in treatments for neurological diseases, anti-inflammatory and analgesic treatment, and soft-tissue injury and rehabilitation therapy, we believe that escin will play a greater role in the prevention and treatment of CVD and will make significant contributions to human health.
